# The Actor, Partner, Similarity Effects of Personality, and Interactions with Gender and Relationship Duration among Chinese Emerging Adults

**DOI:** 10.3389/fpsyg.2017.01698

**Published:** 2017-09-29

**Authors:** Yixin Zhou, Kexin Wang, Shuang Chen, Jianxin Zhang, Mingjie Zhou

**Affiliations:** ^1^CAS Key Laboratory of Mental Health, Institute of Psychology, Chinese Academy of Sciences, Beijing, China; ^2^Department of Psychology, University of Chinese Academy of Sciences, Beijing, China; ^3^School of Journalism and Communication, Tsinghua University, Beijing, China; ^4^Youth Research Institute, China Youth University of Political Studies, Beijing, China

**Keywords:** BFI, relationship quality, actor-partner effect, personality similarity effect, hierarchical linear model

## Abstract

Understanding personality effects and their role in influencing relationship quality, varied according to gender and relationship duration, could help us better understand close relationships. Participants were Chinese dating dyads and were asked to complete both the Big Five Inventory and Perceived Relationship Quality Component scales. Males and those who had a long-term relationship perceived better relationship quality; individuals who scored higher on agreeableness, conscientiousness, openness, and emotional stability enjoyed better relationship quality; gender and/or relationship duration moderated the actor effect of extraversion and the partner effects of conscientiousness, emotional stability, and openness on relationship quality. Regarding the profile similarity, those couples who were more dissimilar in their profile personality had better relationship quality, especially when they were in a relatively long-term relationship. Meanwhile, with an increase in profile similarity, the males' perceived relationship quality decreased.

## Introduction

People are born into a complex network of social relations, of which the intimate relationship is one of the most important. In this kind of relationship, individuals interact with each other directly and deeply to pursue and construct a happy life. A good long-term romantic relationship, in turn, can make an individual happier and lead to longer life expectancy (Claxton et al., 2012).

In explorations of the factors affecting romantic relationship quality at the individual level, the role of personality has been widely confirmed. Personality is a stable and fundamental psychological construct (Donnellan et al., 2005). It better predicts the degree of relationship quality compared to other factors related to romantic relationships—such as attitudes, values, and beliefs (Luo and Klohnen, 2005). Therefore, understanding how personality influences the relationship quality can help scholars better understand the mechanisms of romantic relationship variation.

### Actor effect of personality on relationship quality

People's personalities will first have an impact on their relationship satisfaction. This is called the actor effect. Normally, a good relationship is related to a higher level of relationship expectation and satisfaction, a longer relationship duration, and a less possibility of breakup. Among the Big Five personality traits, emotional stability is often associated with positive relationship expectations. Low levels of emotional stability (high neuroticism) increase relationship instability and the likelihood of breakups (Roberts et al., 2007; Solomon and Jackson, 2014). With the exception of emotional stability, agreeableness has the most stable and strongest effect (Neyer and Voigt, 2004; Watson et al., 2004; Furler et al., 2014; Schaffhuser et al., 2014). Extraversion and conscientiousness also have an influence on adjustment, conflict prevention (Watson et al., 2004), and future levels of satisfaction (Solomon and Jackson, 2014). With regard to openness, it exhibits the weakest and most inconsistent effects (McCrae, 1996). As can be seen, a good relationship is often associated with high levels of emotional stability, agreeableness, extraversion, and conscientiousness.

### Partner effect of personality on relationship quality

The personality is not only related to the formation of the individual's satisfaction but also to the perceived relationship quality of his or her partner; this is called the partner effect (Kenny et al., 2006). A partner's personality affects an individual's evaluation of relationship quality through daily interaction, emotional sharing, and conflict resolution. A meta-analysis of 3,838 subjects among 19 samples demonstrated that, among the Big Five personality traits, an individual's emotional stability has the highest level of prediction of the partner's relationship satisfaction. Emotional stability is followed by agreeableness, conscientiousness, and extraversion (Malouff et al., 2010). However, the results may vary across age cohorts. For example, in younger couples, an individual's openness and agreeableness can directly affect the partner's relationship satisfaction and emotional stability can enhance the perceived sense of security of the partner (Neyer and Voigt, 2004). Therefore, more evidence should be provided to clarify the partner effect of personality in dating relationships.

### Similarity effect of personality on relationship quality

Apart from the effects of individual-level personality, how couples' personalities co-influence the relationship is another key question that researchers have focused on. There are two competing hypotheses with respect to dyadic personality fit and relationship quality: the similarity hypothesis and the complementary hypothesis. The similarity hypothesis poses that similarity in some important areas will make people evaluate other people as more attractive and perceive higher levels of relationship satisfaction (Lucas et al., 2004). This is because similar partners not only confirm the recognition of the world and self but also reduce the risk of conflict. They increase equality of communication and promote mutual understanding (Morry, 2005). In contrast, according to the complementary hypothesis, individuals are more satisfied with people who complement them. The reason complementary partners are more beneficial to the relationship is that those people have a higher possibility of meeting individual needs (De Raad and Doddema-Winsemius, 1992). Complementary couples can learn from each other in order to better face and solve problems. Luo and Klohnen (2005) investigated newlyweds' personality similarities. They found that, although real couples showed no obvious similarities compared to randomly paired samples, similarity was still a good predictor of marital satisfaction. Similar results were also found in dating couples by Gonzaga et al. (2007) when personality similarities among married couples were also reported. Gonzaga et al.'s (2007) longitudinal investigations demonstrated that similarities in personalities and emotions could help newlywed couples maintain good relationship quality. On the other hand, when viewed from a long-term marriage perspective, profile personality similarity has a negative impact on marital satisfaction for middle-aged and older adults (Shiota and Levenson, 2007). In addition, some scholars pointed out that couples with similar and dissimilar personality traits exhibited no differences in their relationship satisfaction (Dyrenforth et al., 2010; Orth, 2013). In general, although more empirical studies found a positive similarity impact on couples' relationship quality, they were still inconsistent in answering whether and how personality similarity improves the relationship quality.

### Gender and its interaction with personality in predicting relationship quality

According to the social role theory (Eagly et al., 2000), gender roles are a reflection of culture and foster sex-differentiated behavior. Females were more connected with communal roles whereas males were more connected with agentic roles. As a result, females tended to evaluate their partners and relationships more positively. A multiple data investigation (Winquist et al., 1998) revealed the existence of the positive bias of the female perceiver, which is named the female positivity effect. The gender difference can further extend to relationship-related factors—such as sexual cognitions (Moyano and Sierra, 2013), preferred characteristics for short-term and long-term partners (Castro and de Araujo Lopes, 2011), mate retention strategies (Holden et al., 2014), and personality. Females are higher in agreeableness but lower in emotional stability and assertiveness (Costa et al., 2001).

Owing to the gap between the meanings of romantic relationship and personality, it is not surprising to see gender asymmetry in personality effects among relationship outcomes. For example, a highly conscientious female will enjoy the relationship more, a highly agreeable man will raise his partner's satisfaction, and not vice versa (Neyer and Voigt, 2004). It should be pointed out that, with the accepted asymmetry view, findings of the interaction between personality and gender still vary across literature (Watson et al., 2000, 2004; Luo and Klohnen, 2005).

### Time variation of personality effects on relationship quality

Another question is in regard to how the patterns of personality effect and relationship quality changed through the course of the relationship.

First, scholars should not ignore the distinct features of different relationship stages. The motivations of dating couples are mainly related to companionship and self-growth, and marriage is motivated by the values of love and family in addition to complex influencers, such as economic situation, family background, and children. Thus, dating and married couples vary in the expectations of communication quality, problem encountering, responsibility attribution, and conflict resolution (Cupach and Metts, 1986; Stafford and Reske, 1990). Comparisons between dating and married couples showed the most consistent effect was conscientiousness. It is more important for both sides of the dating couples, and its impacts decrease when dyads get married (Watson et al., 2000; Holland and Roisman, 2008).

Second, except for the expectation changes when transitioning from short-term to long-term relationships, whether a trait is observable and evaluative will influence when it begins to influence the deepening of the relations (Vazire, 2010). Extraversion, which is related to sociability and overt behavior, can affect mating decisions at the beginning. In speed-dating contexts, men's high extraversion will lead to their popularity (Back et al., 2011). Correspondingly, it takes time for individuals to discover their partners' openness and emotional stability levels due to their low observability features. In a longitudinal study on married couples, Bouchard and Arseneault (2005) observed the emergence of negative impact of women's openness and low emotional stability levels on the relationship.

In addition, couples with different personality patterns prefer distinct relationship-related strategies for issues, such as how to be involved as lovers (Barelds and Barelds-Dijkstra, 2007) and how to maintain romantic relationships (Holden et al., 2014). When meeting partners complementary in extraversion, emotional stability, and openness, people are more likely to fall in love at first sight whereas conscientiousness similarity is relatively higher in friends-first relations (Barelds and Barelds-Dijkstra, 2007).

Thus, when thinking about the personality effects on relationship quality, scholars should take the relationship duration into consideration as well.

### The measurement indices of personality (dis)similarity

In studies with a focus on how couples' personalities interact with each other, the impact of measurement methodology cannot be ignored. Two issues should be focused on: one is how to define personality similarity and difference, and the other is how to determine which kind of measurement is more accurate in describing personality similarities and differences. When thinking about the definition of personality similarity (or low complementarity), early researchers focused on the differences between discrete personality traits (Watson et al., 2004; Holland and Roisman, 2008); this is called the variable-centered approach. However, the various characteristics of one individual should be regarded as a whole. Further, the two sides of a couple constitute a complete system. The comparison of a particular personality trait can tell scholars how its differences affect the couple's relationship quality, but it cannot answer whether a couple with particular patterns is able to achieve a better relationship quality (Luo and Klohnen, 2005). Studies (Luo and Klohnen, 2005; Gaunt, 2006) have revealed that, compared to similarities in a single trait, profile personality similarity (called the couple-centered approach) has a stronger and more consistent influence capacity toward marital satisfaction. Therefore, in this study, we intended to explore how a couple's profile personality similarity affects relationship quality.

Regarding the issue of which similarity index is more accurate in reflecting the similarities and differences of personality traits, previous studies (Watson et al., 2000; Gaunt, 2006; Claxton et al., 2012) primarily used correlations and absolute scores to evaluate the two sides of one couple in a single personality trait. This approach was criticized for obscureness regarding deeper interaction mechanisms (Malouff et al., 2010). McCrae (2008) compared four personality similarity indices: the Pearson correlation, the Cattel correlation, the McCrae correlation, and the double-entry intraclass correlation. The results demonstrated that the intraclass correlation coefficient (ICC) was efficient and accurate in distinguishing matched and unmatched individuals and in describing the similarity and difference of a couple's personality, both at the factor level and the facet level. When the personality scores of one side were extremely high and the scores of the other side were extremely low but their trends were similar, the ICC was still very sensitive but the common correlation was not able to detect the large difference between their personality traits. Therefore, in this study, the ICC was selected to reflect the overall similarity of personality traits among couples. We included multiple similarity indices simultaneously in the analysis to compare the differences caused by measurement methods.

Previous researchers have rarely considered the role of the actor-partner model and profile similarity at the same time. However, controlling individual factors under the analysis of profile similarity is necessary. Collecting samples from Australia, Britain, and Germany, Dyrenforth et al. (2010) used hierarchical linear models to analyze the actor and partner effects of individual-level personality and couple-level profile similarity on relationship satisfaction. They also compared three indices of profile similarity: the average absolute difference, the ICC of raw scores, and the ICC of standardized scores. The results indicated that the Big Five personality traits all illustrated significant actor effects. Agreeableness, conscientiousness, and emotional stability have significant partner effects. The effect sizes of the profile similarity indices were small and weak after controlling for actor and partner effects.

### The current study

Previous studies have mostly emphasized marriage relationships, but the mechanisms in marriage relationships contrast with those of dating couples. Apart from the time variation in personality effects, the assortative personalities of dating couples are often the results of mutual selection; that is, individuals fall in love because they are (dis)similar at that moment. The matching of married couples' personalities cannot escape the convergence effect; that is, couples become more similar with time (Gonzaga et al., 2007). Therefore, it is necessary to test the influence mechanism of dating couples' relationship qualities with the investigation on relationship duration.

Second, Chinese emerging adults are unique with regard to romantic relationships (Tang and Zuo, 2000; Gao, 2001; Ng and Cheng, 2010; Chan et al., 2012). Chinese people have lower degrees of desire for pleasure and passion as well as higher degrees of intimacy, commitment, relationship constraints, and mutual respect in romantic relationships. They depend more on the relationship. They are closer to each other psychologically. Therefore, it is of special significance to conduct a study that applies to young Chinese couples.

The literature review suggests that most of the researchers support the statement that the interactions between the couples' personality traits and their similarities have a crucial influence on relationship quality. However, whether from the actor or the partner perspective and whether based on single traits or profile similarity, studies have not yet given uniform and clear results. At the same time, the gender difference in close relationships should also not be neglected.

Based on this, we tried to follow the design of Dyrenforth et al. (2010). Using the method of a hierarchical linear model, we set out with the aim of examining the actor, partner, similarity effects of personality, and the interactions between personality effects, gender and relationship duration among Chinese emerging adults. Related material is available on the Open Science Framework at https://osf.io/3f6pv/.

## Method

### Participants and procedure

We performed a priori power analysis using the program G^*^power 3.1 (Friedkin, 2011). Given the Dyrenforth et al. (2010)'s findings where an observed total *R*^2^ of actor, partner and similarity personality effects was 0.085 for a sample of over 5,278 individuals, the max sample size required for our models was 542 with a Type I error rate of α = 0.05.

Two hundred eighty-one pairs of dating couples from five universities in Shanghai, Beijing, Guangdong, Hebei, and Henan in China were collected. The mean age of the participants was 21 (*SD* = 2.29). Their relationship durations were measured by a 7-point scale: 1 = *below 3 months*, 2 = *3–6 months*, 3 = *6–12 months*, 4 = *1–2 years*, 5 = *2–3 years*, 6 = *3–4 years*, 7 = *above 4 years*. The mean duration score was 3.73 (*SD* = 1.73). Both partners were asked to independently answer the questionnaire. Then every participant received a gift valuing renminbi (RMB) 15 yuan for each participation.

### Measures

#### Personality

We used the 20-item International Personality Item Pool-Five-Factor Model (Mini-IPIP) designed by Donnellan et al. (2006). It contains five dimensions of extraversion, openness, agreeableness, conscientiousness and emotional stability. Each dimension has four 5-point items. Li et al. (2012) reported good reliability and validity among the Chinese population. In the present study, the confirmatory factor analysis showed a moderate construct validity [$\chi_{\left(135\right)}^{2}$ = 440.135, GFI = 0.923, RMSEA = 0.063]. Cronbach's α coefficients were 0.61 for extraversion, 0.60 for agreeableness, 0.61 for conscientiousness, 0.64 for emotional stability, and 0.62 for openness. We believe that the relatively low reliability was due to the high homogeneity of personality traits in our sample (all participants were college students). The relative mean tiem variance index was 0.49, 0.36, 0.44, and 0.29 for five dimensions comparing to the riginal sample (Donnellan et al., 2006), which means this sample is more homogeneous than the comparison sample. That could lead to a small variance and low reliability. Onwuegbuzie and Daniel (2002) suggested that the data does not need to be abandoned under such circumstances.

Personality similarity was represented by trait discrepancy and profile similarity. Trait discrepancy was measured by the absolute difference score between both dyads of the couple. A greater absolute score means a higher personality discrepancy. The profile similarity of the couple's personality traits was specified using two indices: average discrepancy and ICC score. Following Dyrenforth et al. (2010), we first averaged the discrepancy score of five personality traits, named as the couple's average discrepancy; second, we calculated the standardized ICC of the personality trait scores of the couple. This is also called global similarity. We computed the individual trait-wise z-score by grand mean and standard deviation then calculated the ICC of these standardized scores for each couple. The standardization manipulation can filter out measurement errors, such as stereotype effect (Kenny and Acitelli, 1994) and normativeness (Allik et al., 2015).

#### Relationship quality

The six-item perceived relationship quality component (Fletcher et al., 2000) was used to measure six aspects of relationship quality: satisfaction, love, commitment, trust, intimacy, and passion on a 7-point Likert scale. The Cronbach's α was 0.91. This scale was applied to the Chinese samples previously (Ng and Cheng, 2010). We performed a one-factor confirmatory factor analysis. The results confirmed that the construct validity was good among the Chinese emerging adult sample [$\chi_{\left(9\right)}^{2}$ = 30.216, GFI = 0.976, RMSEA = 0.076 < 0.1].

#### Data analysis strategies

The hierarchical linear model is suitable for exploring the couples' data when the individuals in the same cluster are more alike than cross-cluster individuals and makes a better control of intra- and cross-level variance at the same time (Wendorf, 2002). In the model, the individual-level data consisted of gender and the actors' and partners' Big Five traits. The couple-level data, for which scholars measured couples as a unit, included relationship duration as well as trait and profile similarity indices. Further, bootstrap confidence interval analysis was used to ensure the robustness of the estimation. The data analysis was conducted using package lme4 (Bates et al., 2015) in software R (R Core Team, 2017).

Following the analysis strategies of Dyrenforth et al. (2010), we performed the analysis in two steps. In the first step, we examined the relationship between trait-level discrepancy and relationship quality. We performed a two-level linear analysis separately for each personality trait. At the individual level, we included gender (Male = –0.5, Female = 0.5) as well as actor and partner effects of one dimension of personality traits. At the couple level, we included the trait discrepancy score and relationship duration. In addition, two-way and three-way interactions among gender, duration, and personality effects (actor, partner, trait discrepancy effects) were also included. Variables were grand-centered when they were included in the model.

In the second step, we aimed to determine the relationship between profile-level similarity and relationship quality. In Model 1 (M1), we only considered the effects of gender, duration, and the profile similarity by adding the interactions between these three variables. On the basis of M1, Model 2 (M2) added the actor and partner effects of the Big Five personality traits, and the interactions with gender and duration. In addition, we applied the two indices of profile similarity (average discrepancy, ICC), respectively in these two models to compare the differences and to achieve a more reliable result.

## Results

The results of descriptive and correlation analysis are shown in Table 1. To compare the individual level result by gender, we did correlation separately. The intercorrelations for males were in the lower diagonal of the table; for females, they were in the upper diagonal. Males' relationship qualities were positively related to self-agreeableness, conscientiousness, openness, emotional stability, and partners' conscientiousness level (|*r|*s > 0.12, *p* < 0.05). Females' relationship qualities were related to self-extraversion, agreeableness, openness, and emotional stability level as well as partner's agreeableness, conscientiousness, and emotional stability (*|r|*s > 0.12, *p* < 0.05). The couple-level correlation revealed that the indices describing the profile similarity of the personality traits were statistically related (*r* = −0.64, *p* < 0.001). Couples' personality similarities were stable with the passing of time (|*r|*s < 0.08, *p* > 0.05).

**Table 1:** Correlations analysis among gender, duration, personality traits and profile similarity indices.

	***M***	***SD***	**1**	**2**	**3**	**4**	**5**	**6**	**7**	**8**	**9**	**10**	**11**
**INDIVIDUAL LEVEL (N = 562)**													
1. Actor extraversion	3.00	0.64	1	0.24^***^	0.19^**^	0.15^*^	0.21^***^	0.10	0.04	0.03	−0.03	0.08	0.21^***^
2. Actor agreeableness	3.63	0.55	0.19^**^	1	0.29^***^	0.12^*^	0.17^**^	0.03	0.19^**^	0.10	−0.01	0.12^*^	0.24^***^
3. Actor conscientiousness	3.52	0.58	0.16^**^	0.33^***^	1	0.10	0.15^*^	−0.12^*^	−0.02	0.16^**^	0.02	0.09	0.04
4. Actor openness	3.41	0.64	0.16^**^	0.20^**^	0.21^***^	1	−0.02	−0.01	−0.04	0.06	0.12^*^	0.08	0.12^*^
5. Actor emotional stability	3.17	0.67	0.20^**^	0.10	0.24^***^	0.15^*^	1	0.07	0.08	0.15^*^	0.05	0.13^*^	0.13^*^
6. Partner extraversion	3.00	0.64	0.01	0.04	0.03	−0.03	0.08	1	0.19^**^	0.16^*^	0.16^**^	0.20^**^	0.04
7. Partner agreeableness	3.63	0.55	0.03	0.19^**^	0.10	−0.01	0.12^*^	0.24^***^	1	0.33^***^	0.20^**^	0.10	0.15^*^
8. Partner conscientiousness	3.52	0.58	−0.12^*^	−0.02	0.16^*^	0.01	0.09	0.19^**^	0.29^***^	1	0.21^***^	0.24^***^	0.17^**^
9. Partner openness	3.41	0.64	−0.01	−0.04	0.06	0.12^*^	0.08	0.15^*^	0.12^*^	0.10	1	0.15^*^	0.12
10. Partner emotional stability	3.17	0.67	0.07	0.08	0.15^*^	0.05	0.13^*^	0.21^***^	0.17^**^	0.15^*^	−0.02	1	0.13^*^
11. Relationship quality	5.65	0.97	0.06	0.20^**^	0.13^*^	0.19^**^	0.17^**^	0.05	0.07	−0.01	0.02	−0.04	1
**COUPLE LEVEL (N = 281)**													
1. Duration	3.72	1.73	1										
2. Extraversion discrepancy	0.66	0.56	0.04	1									
3. Agreeableness discrepancy	0.54	0.47	0.03	0.11	1								
4. Conscientiousness discrepancy	0.58	0.54	−0.01	0.25^***^	0.16^**^	1							
5. Openness discrepancy	0.65	0.54	0.08	0.07	0.10	0.18^**^	1						
6. Emotional stability discrepancy	0.69	0.56	0.06	0.10	0.17^**^	0.21^**^	0.16^**^	1					
7. Average discrepancy	0.62	0.29	0.07	0.55^***^	0.51^***^	0.61^***^^*^	0.55^***^	0.60^***^	1				
8. ICC	−0.09	0.54	−0.05	−0.42^***^	−0.27^***^	−0.37^***^	−0.36^***^	−0.39^***^	−0.64^***^	1			

*The intercorrelations of the individual level variables for males were in the lower diagonal, for females were in the upper diagonal*.

* *p < 0.05*,

** *p < 0.01*,

*** *p < 0.001*.

We first examined the effect of individual personality traits on relationship quality. In the zero model, the ICC was 0.49; thus the hierarchical linear model was suggested for analysis. The results are shown in Table 2. The main effect of gender was significant (*β*_*mean*_ = −0.25, *p*s < 0.001). Males had higher relationship qualities than females. The tendency of the duration effect was positive (*β*_*mean*_ = 0.09, *p*s < 0.07). With time, the couples tended to achieve better relationship qualities.

**Table 2:** The regression coefficient of personality effect on relationship quality.

**Effects**	**Extraversion**				**Agreeableness**				**Conscientiousness**				**Emotional stability**				**Openness**			
	**β**	**SE**	***p***	**95%CI**	**β**	**SE**	***p***	**95%CI**	**β**	**SE**	***p***	**95%CI**	**β**	**SE**	***p***	**95%CI**	**β**	**SE**	***p***	**95%CI**
Gender	−0.24	0.06	0.00	−0.36, −0.13	−0.29	0.06	0.00	−0.41, −0.18	−0.25	0.06	0.00	−0.36, −0.14	−0.22	0.06	0.00	−0.34, −0.10	−0.25	0.06	0.00	−0.36, −0.13
Duration	0.08	0.03	0.01	0.01, 0.15	0.06	0.03	0.07	−0.000.12	0.09	0.03	0.01	0.02, 0.15	0.09	0.03	0.01	0.02, 0.15	0.06	0.03	0.05	+0.000.13
Gender × Duration	−0.03	0.03	0.36	−0.1, 0.03	−0.04	0.03	0.23	−0.11, 0.02	−0.03	0.03	0.31	−0.1, 0.03	−0.02	0.03	0.61	−0.08, 0.05	−0.02	0.03	0.60	−0.09, 0.04
Actor	0.10	0.09	0.29	−0.09, 0.28	0.36	0.11	0.00	0.15, 0.56	0.21	0.10	0.03	0.01, 0.39	0.24	0.09	0.01	0.07, 0.41	0.31	0.09	0.00	0.14, 0.48
Actor × Gender	0.24	0.13	0.07	−0.03, 0.52	0.01	0.16	0.94	−0.32, 0.30	−0.18	0.15	0.22	−0.49, 0.12	−0.06	0.13	0.64	−0.31, 0.2	−0.15	0.13	0.27	−0.38, 0.10
Actor × Duration	−0.08	0.06	0.17	−0.20, 0.03	−0.05	0.06	0.44	−0.17, 0.08	−0.02	0.06	0.71	−0.13, 0.1	0.00	0.05	0.96	−0.11, 0.12	−0.02	0.05	0.68	−0.13, 0.09
Actor × Gender × Duration	0.18	0.08	0.02	0.03, 0.35	0.11	0.10	0.26	−0.09, 0.32	0.18	0.09	0.05	−0.01, 0.35	0.07	0.08	0.38	−0.09, 0.24	0.03	0.08	0.73	−0.14, 0.18
Partner	0.06	0.09	0.51	−0.13, 0.24	0.00	0.11	0.99	−0.22, 0.21	−0.06	0.11	0.58	−0.26, 0.15	−0.08	0.09	0.38	−0.25, 0.11	−0.03	0.09	0.77	−0.21, 0.16
Partner × Gender	−0.04	0.13	0.75	−0.31, 0.22	0.24	0.16	0.14	−0.09, 0.56	0.32	0.15	0.03	0.03, 0.60	0.24	0.13	0.06	+0.00, 0.48	0.21	0.13	0.12	−0.04, 0.46
Partner × Duration	0.03	0.05	0.52	−0.06, 0.14	0.05	0.07	0.44	−0.08, 0.21	0.19	0.06	0.00	0.08, 0.30	0.13	0.05	0.02	0.02, 0.23	0.13	0.05	0.02	0.03, 0.24
Partner × Gender × Duration	−0.14	0.08	0.08	−0.31, 0.01	−0.10	0.10	0.27	−0.3, 0.09	−0.15	0.09	0.09	−0.33, 0.03	−0.19	0.08	0.02	−0.34, −0.02	−0.12	0.08	0.13	−0.29, 0.04
Discrepancy	0.19	0.11	0.08	−0.03, 0.42	0.19	0.12	0.13	−0.06, 0.44	0.10	0.12	0.43	−0.16, 0.33	0.07	0.10	0.53	−0.14, 0.27	0.24	0.11	0.02	0.02, 0.46
Discrepancy × Gender	−0.16	0.11	0.15	−0.37, 0.06	0.00	0.13	0.98	−0.26, 0.25	−0.03	0.12	0.81	−0.28, 0.20	−0.04	0.10	0.73	−0.24, 0.16	−0.08	0.11	0.44	−0.28, 0.14
Discrepancy × Duration	0.06	0.07	0.39	−0.08, 0.19	0.05	0.07	0.48	−0.09, 0.2	0.15	0.07	0.04	0.01, 0.29	0.07	0.07	0.31	−0.07, 0.19	0.09	0.06	0.15	−0.04, 0.22
Discrepancy × Gender × Duration	−0.13	0.07	0.05	−0.26, 0.01	0.03	0.07	0.70	−0.11, 0.17	−0.10	0.07	0.17	−0.25, 0.04	−0.01	0.07	0.93	−0.13, 0.13	−0.11	0.06	0.09	−0.24, 0.02
*R*^2^	0.09				0.09				0.08				0.09				0.10			

*Unstandardized regression*.

*95% BCI refers to bootstrapped confidence intervals*.

**Figure 1:**
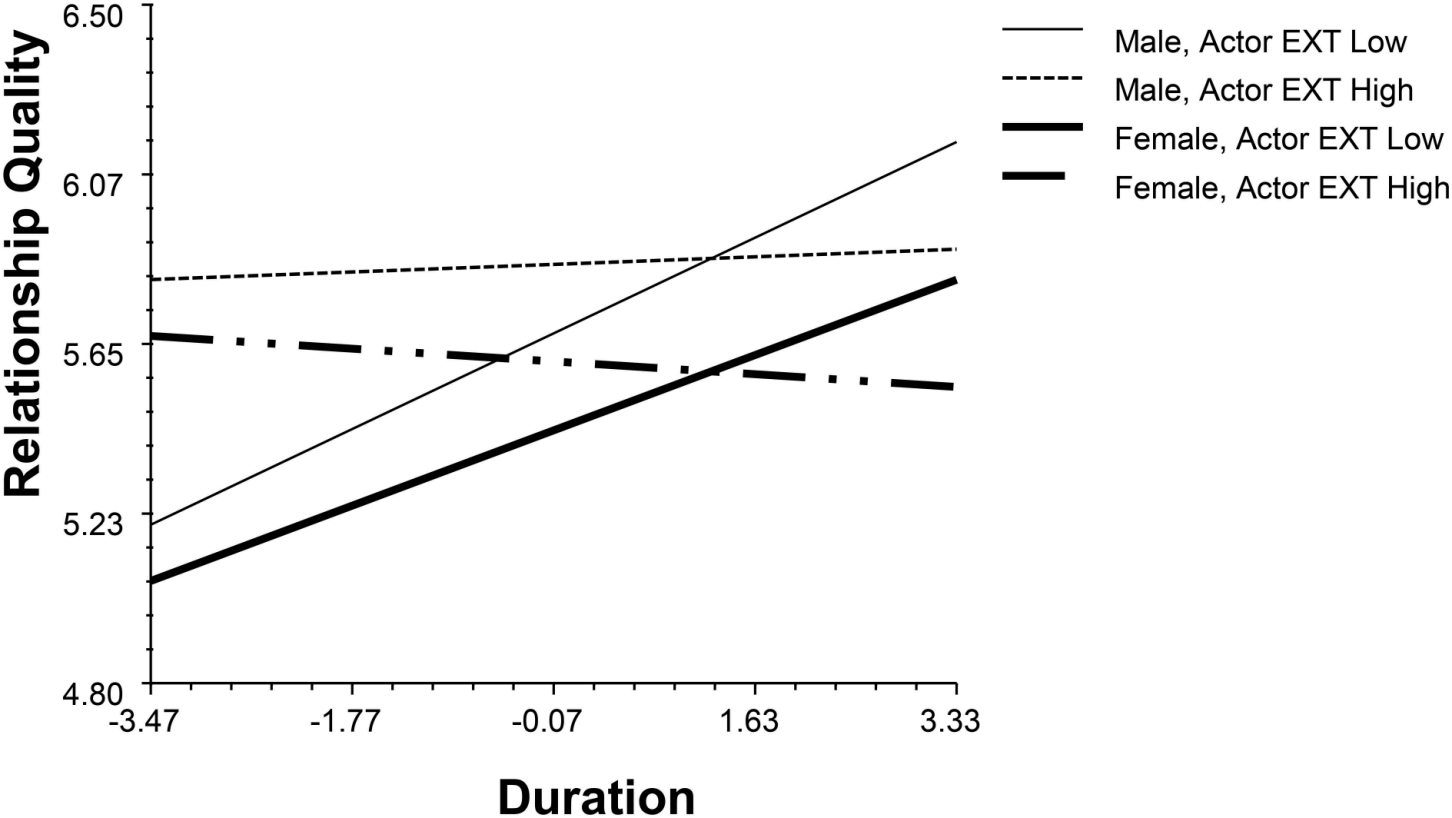
The interaction between actor's extraversion, gender and duration.

Regarding actor effects, agreeableness (AGR), conscientiousness (CON), emotional stability (ES), and openness (OPN) positively associated with relationship quality (*β*_*AGR*_ = 0.36, *p* < 0.001, 95% BCI: 0.15,0.56; *β*_*CON*_ = 0.21, *p* = 0.03, 95% BCI: 0.01, 0.39; *β*_*ES*_ = 0.24, *p* = 0.01, 95% BCI: 0.07, 0.41; *β*_*OPN*_ = 0.31, *p* < 0.001, 95% BCI: 0.14, 0.48). The extraversion (EXT) actor effect interacts with gender and duration (*β*_*EXT*_ = 0.18, *p* = 0.02, 95% BCI: 0.03, 0.35, see Figure 1). Although, introvert had worse initial relationship qualities, this disadvantage weakened with the increase of the time they engaged in the relationship.

**Figure 2:**
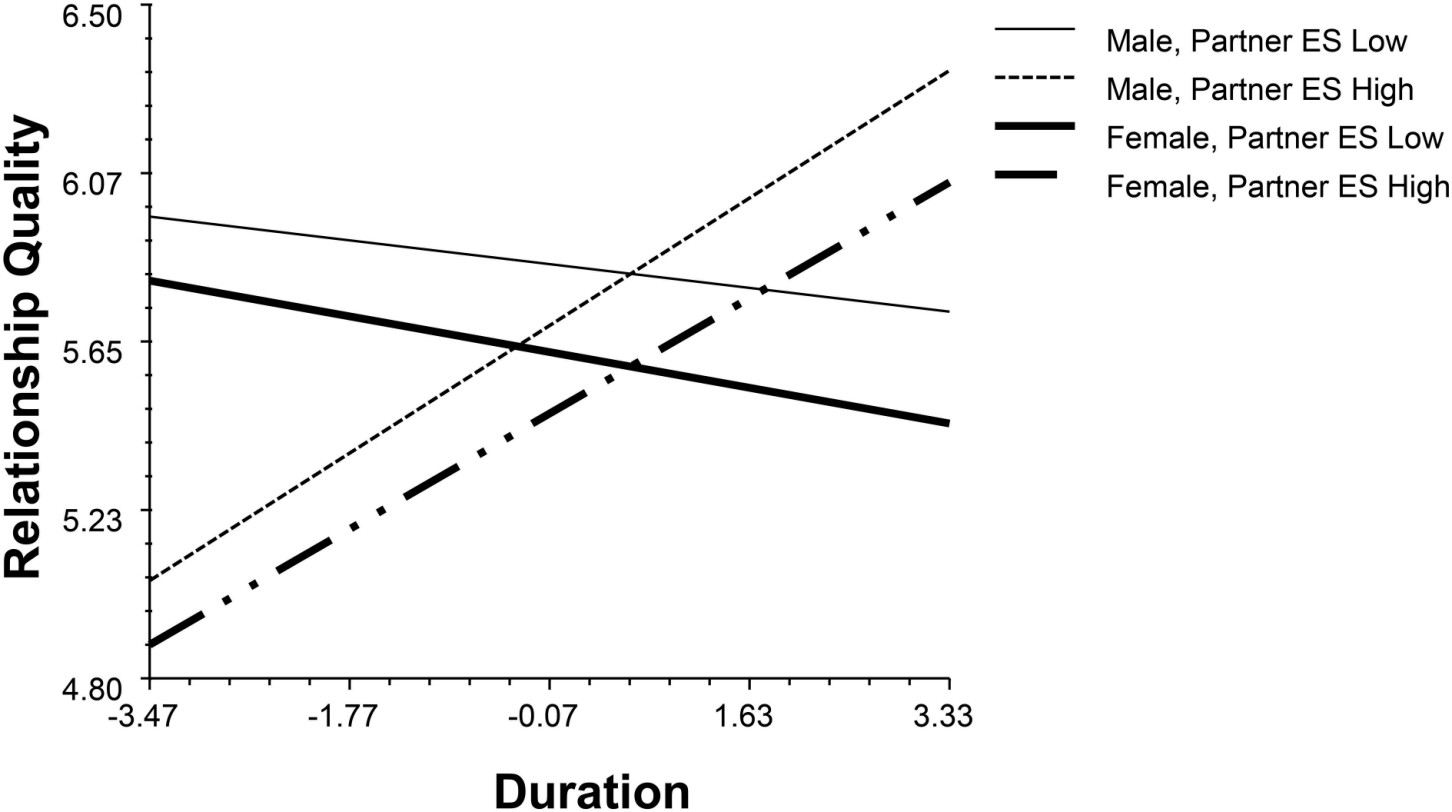
The interaction between partner's emotional stability, gender and duration.

**Figure 3:**
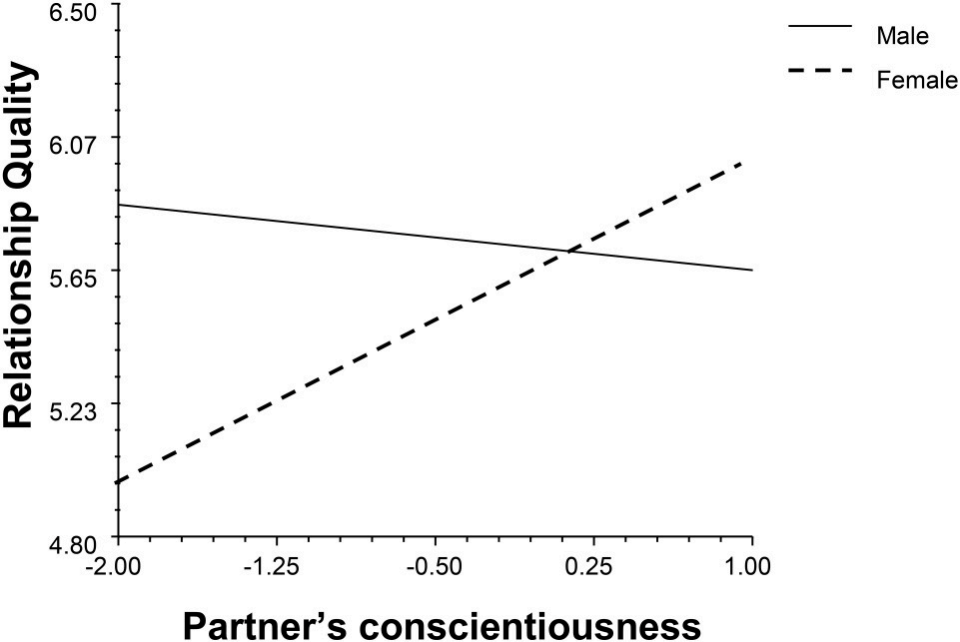
The interaction between partner's conscientiousness and gender.

**Figure 4:**
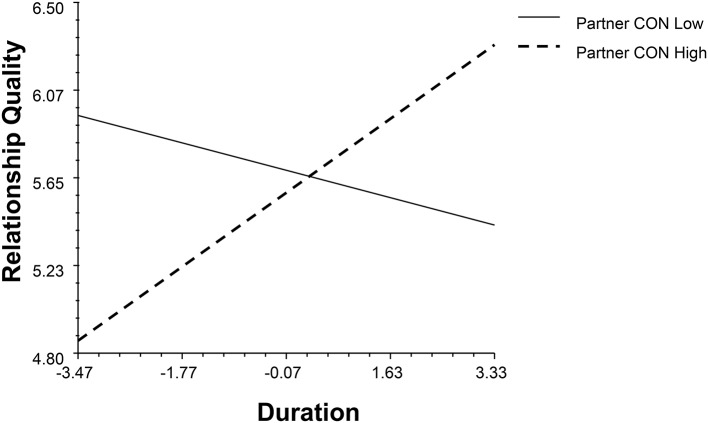
The interaction between partner's conscientiousness and duration.

**Figure 5:**
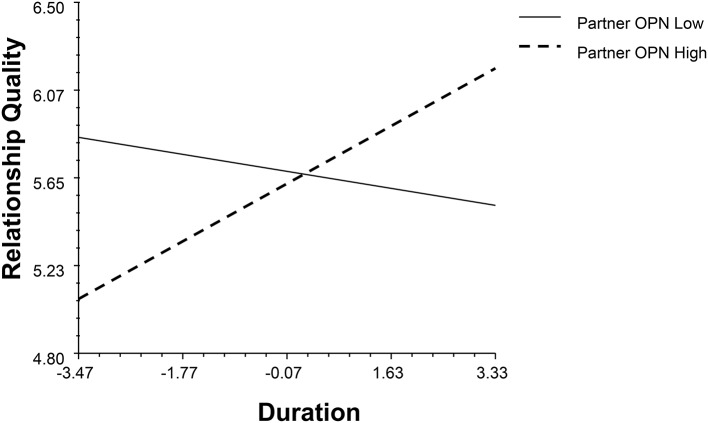
The interaction between partner's openness and duration.

The partner effect of emotional stability moderated with gender and duration (*β* = −0.19, *p* = 0.02, 95% BCI: −0.34, −0.02, see Figure 2). When people with partners with low emotional stability stabilized the satisfaction with time, people with partners with high emotional stability positively associated with their relationship quality. The partner's conscientiousness (CON) moderated with gender and duration independently (*β*_1_ = 0.32, *p* = 0.03, 95% BCI: 0.03, 0.60; *β*_2_ = 0.19, *p* < 0.001, 95% BCI: 0.08, 0.30, see Figures 3, 4). High levels of partner conscientiousness was detrimental for males' satisfaction but beneficial for that of females. The pattern of interaction between duration and partner openness was reflected in the interaction between duration and partner conscientiousness (*β*_*OPM*_ = 0.13, *p* = 0.02, 95% BCI: 0.03, 0.24); low levels of partner openness/conscientiousness is positively associated with short-term relationships whereas high levels of partner openness/conscientiousness is more important for long-term relationships (see Figures 4, 5).

**Figure 6:**
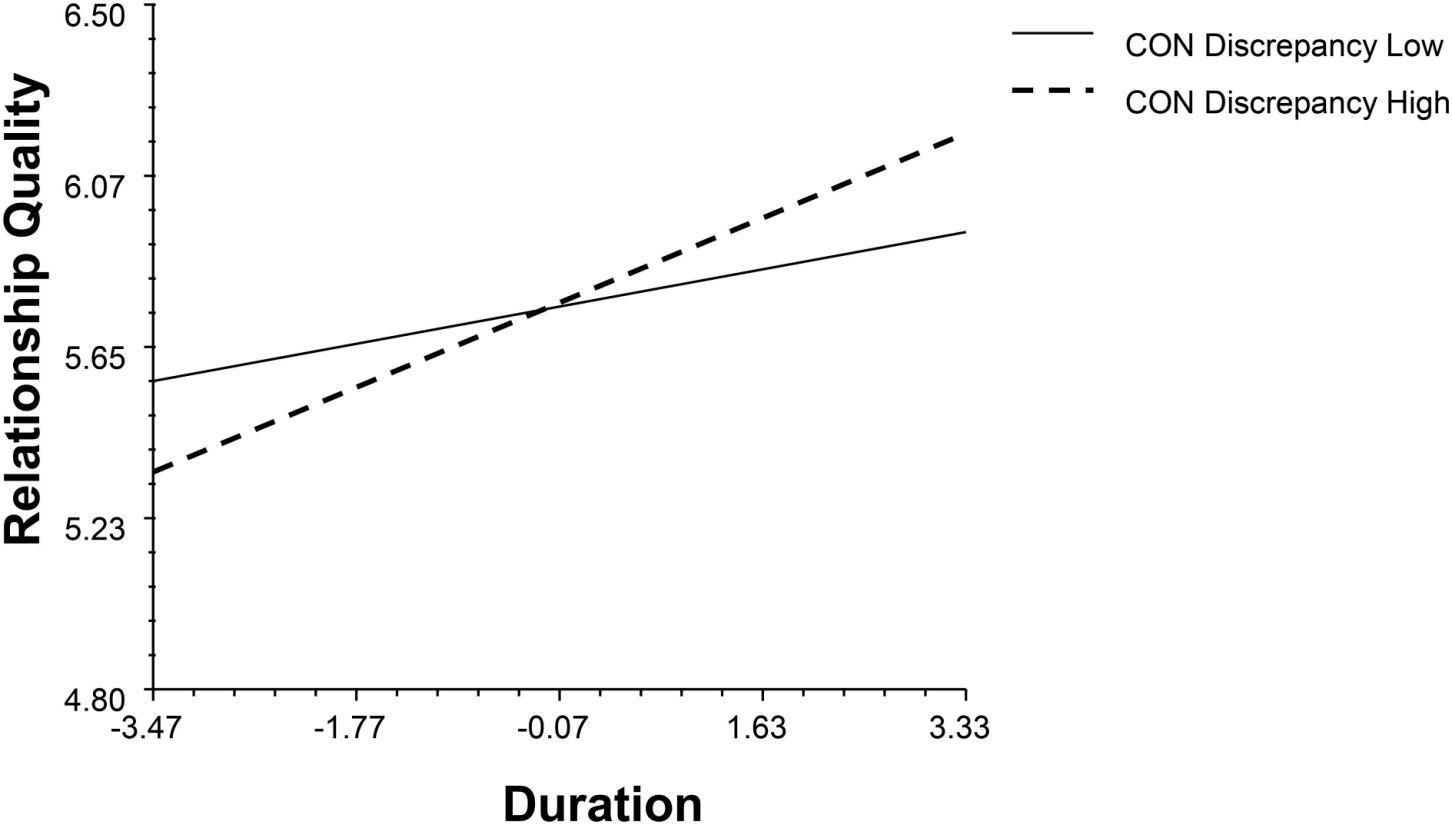
The interaction between conscientiousness discrepancy and duration.

The couple-level analysis of trait discrepancy revealed a positive effect of openness discrepancy (*β* = 0.24, *p* = 0.02, 95% BCI: 0.02, 0.46). There are also significant effects on conscientiousness discrepancy with duration (*β* = 0.15, *p* = 0.04, 95% BCI: 0.01, 0.29, see Figure 6). Males in the extraversion complementary dyads had a better relationship quality. The positive role of dissimilarity in conscientiousness increases over time.

**Figure 7:**
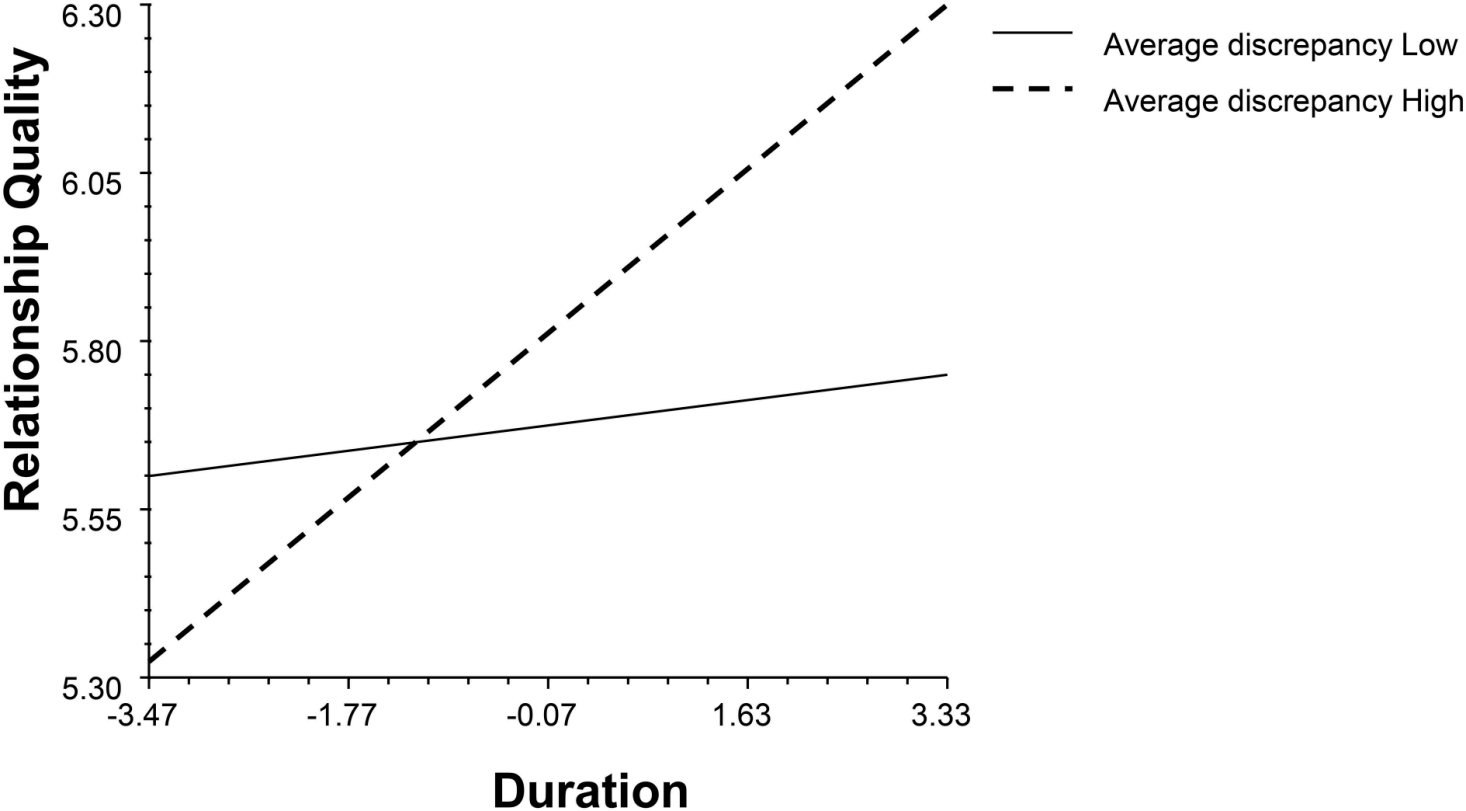
The interaction between average discrepancy and duration (M2).

**Figure 8:**
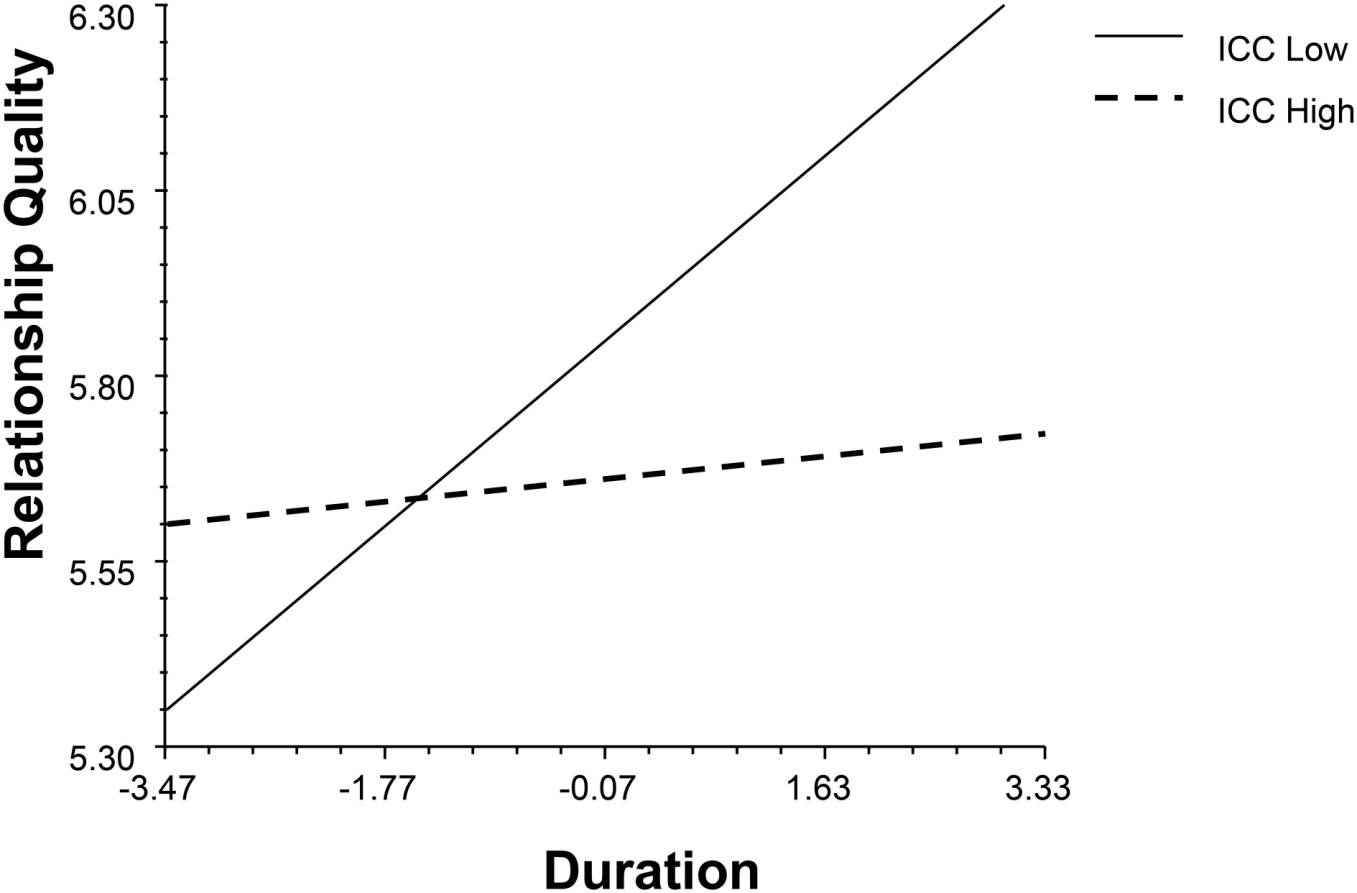
The interaction between ICC and duration (M2).

**Figure 9:**
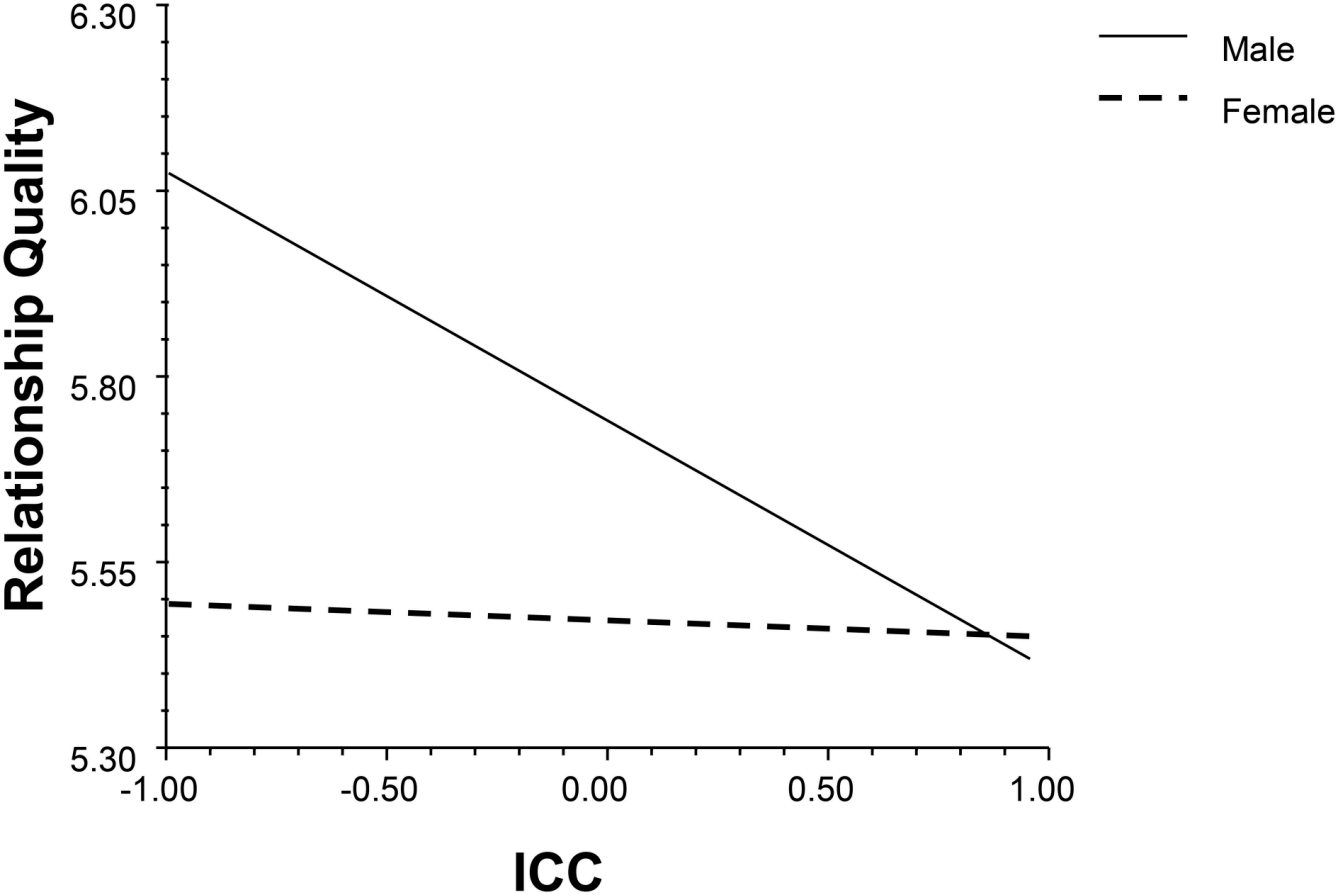
The interaction between ICC and gender (M2).

Second, two models (see Data Analysis Strategies) were used to study the impact of profile similarity on relationship quality. The results are shown in Table 3. The average discrepancy and ICC had main effects on relationship quality (*β*_*Average discrepancyM*1_ = 0.50, *p* = 0.01, 95% BCI: 0.10, 0.90; *β*_*Average discrepancyM*2_ = 0.51, *p* = 0.01, 95% BCI: 0.12, 0.89; *β*_*ICCM*1_ = −0.31, *p* < 0.001, 95% BCI: −0.51, −0.10; *β*_*ICCM*2_ = −0.28, *p* = 0.01, 95% BCI: −0.49, −0.08). Also, profile similarity indices interacted with duration and gender (see Figures 7–9). Individuals in dissimilar dyads tended to achieve better relationship qualities than those in similar dyads, and the satisfaction gap between the similar and dissimilar dyads became wider with the course of time (*β*_*Average discrepancyM*1_ = 0.26, *p* = 0.04, 95% BCI: 0.04, 0.51; *β*_*Average discrepancyM*2_ = 0.35, *p* = 0.01, 95% BCI: 0.07, 0.59; *β*_*ICCM*1_ = −0.14, *p* = 0.03, 95% BCI: −0.26, −0.02; *β*_*ICCM*2_ = −0.16, *p* = 0.01, 95% BCI: −0.29, −0.03). In terms of the gender differences, discrepancy relatively raised males' satisfaction (*β*_*ICCM*1_ = 0.22, *p* = 0.04, 95% BCI: 0.02, 0.42; *β*_*ICCM*2_ = 0.28, *p* = 0.01, 95% BCI: 0.06, 0.48).

**Table 3:** The regression coefficient of profile personality similarity effect on relationship quality.

	**Average discrepancy**								**ICC**							
	**M1**				**M2**				**M1**				**M2**			
	**β**	**SE**	***p***	**95%CI**	**β**	**SE**	***p***	**95%CI**	**β**	**SE**	***p***	**95%CI**	**β**	**SE**	***p***	**95%CI**
Gender	−0.24	0.06	0.00	−0.35, −0.13	−0.26	0.06	0.00	−0.38, −0.15	−0.25	0.06	0.00	−0.36, −0.13	−0.26	0.06	0.00	−0.38, −0.15
Duration	0.07	0.03	0.03	0.01, 0.14	0.09	0.04	0.01	0.01, 0.16	0.07	0.03	0.03	0.01, 0.14	0.08	0.04	0.02	0.01, 0.15
Gender × Duration	−0.02	0.03	0.47	−0.09, 0.05	−0.03	0.04	0.34	−0.11, 0.04	−0.02	0.03	0.52	−0.09, 0.04	−0.03	0.04	0.40	−0.10, 0.04
Profile similarity	0.50	0.20	0.01	0.10, 0.90	0.51	0.20	0.01	0.12, 0.89	−0.31	0.11	0.00	−0.51, −0.10	−0.28	0.11	0.01	−0.49, −0.08
Profile similarity × Gender	−0.20	0.19	0.30	−0.61, 0.18	−0.34	0.20	0.09	−0.78, 0.07	0.22	0.11	0.04	0.02, 0.42	0.28	0.11	0.01	0.06, 0.48
Profile similarity × Duration	0.26	0.13	0.04	0.04, 0.51	0.35	0.13	0.01	0.07, 0.59	−0.14	0.06	0.03	−0.26, −0.02	−0.16	0.06	0.01	−0.29, −0.03
Profile similarity × Gender × Duration	−0.21	0.13	0.09	−0.44, 0.03	−0.18	0.14	0.17	−0.47, 0.08	0.10	0.06	0.11	−0.02, 0.23	0.12	0.07	0.06	−0.01, 0.25
*R^2^*	0.05				0.20				0.05				0.20			

*M1, only includes the variables shown in the table*.

*M2, includes the actor, partner effects of five personality traits, and their two-step, three-step interactions with gender and duration. But those variables are not shown in the table*.

## Discussion

### Gender effect

The results showed that males and females varied in their perceptions of relationship quality; males reported higher relationship qualities than females did. Also, gender generally moderated most of the personality effects.

This is in contrast with previous findings (Winquist et al., 1998; Eagly et al., 2000) that claimed that females tend to regard their relationships more positively than males. At the same time, the phenomenon of females' negative relationship perception was consistently found in the Chinese context (Chen, 2011; Xiao, 2012) and in cross-cultural meta-analyses (Jackson et al., 2014), which leads us to consider the distinctiveness of Chinese culture. On the one hand, because of the one-child policy initiated from 1979 and discrimination against girls in traditional Chinese society, contemporary China has faced a severe shortage of girls (Banister, 2004). On the other hand, the shift to market socialism brought dramatic changes to women's social status (West et al., 1999) and an adaption of young generations to a mixed culture combining both tradition and modernization. These factors jointly affect China's dating and marriage market. Males are pushed to a passive position. Success in competition for partners results in generally better perceptions of the relationships. Moreover, committed Chinese women tend to associate love with negative feelings (Ma et al., 2015), making their perceptions of relationship quality generally lower than that of their partners.

Further, our results showed that gender moderated the connection between an individual's relationship and partner's conscientiousness. The lower her partner's conscientiousness is, the lower a female will evaluate the relationship. Conscientiousness is related to dutifulness, competence, and achievement striving (Costa et al., 2001). Under the social role theory, males are expected by partners to have high levels of resource acquisition ability and faithfulness to the relationship (Eagly et al., 2000). Therefore, male with high conscientiousness is more likely to have a satisfied partner.

### Actor effects of personality on relationship quality

Our study suggested significant positive actor effects for agreeableness, conscientiousness, emotional stability and openness. This accords with the results observed in earlier studies (Barelds, 2005; Malouff et al., 2010). Extraversion interacted with gender and duration. Extraverts' relationship perceptions were relatively high and stable whereas introverts' good relationship perceptions arose through time. Due to sociability and expressiveness, the advantage of extraverts in the initial stages of relationships is supported by previous studies (Vazire, 2010; Back et al., 2011). Being thoughtful and contemplative, introverts were found to take the slow penetration strategy rather than other strategies more often to develop relations (Nelson and Thorne, 2012). Adding the propensity to overestimate the negative affect of positive and pleasant events (Lucas and Diener, 2001; Uziel, 2006), it is reasonable that they gradually begin to enjoy the romantic relationships they are engaged in.

### Partner effect of personality on relationship quality

The main effects of partners' personality traits were weak, which accords with the results of previous studies (Neyer and Voigt, 2004) with the concern that partner effects are smaller and relatively less frequent. However, our study found significant interactions with gender and duration. Generally, the positive aspect of partners' personalities gradually improved the satisfaction with the further deepening of the connection. In the Chinese mate selection market, physical attributes and socioeconomic status were strongly prioritized (Lange et al., 2014). Personal characteristics only gain their emphasis with the deepening of the involvement level (Castro and de Araujo Lopes, 2011). In the meanwhile, time is needed to give insight to the deeper side of the partner and experience the benefits of the partner's trait-related behavior.

Specific to different traits, partners' bright personalities—high levels of conscientiousness, emotional stability, and openness—were highlighted. Partners' openness increased their impact over time for both males and females. In addition, at the beginning of the relationship, individuals with low conscientiousness and emotionally stable partners are more satisfied with the relationships. This is reasonable, considering that the agentic aspect reflected by ignoring rules and being passionate is attractive to strangers (Carter et al., 2014). However, this advantage turns into a disadvantage in the long term (Inancsi et al., 2015).

### Similarity effect of personality on relationship quality

In this study, the couple-level conscientiousness and openness discrepancy exerted a compensation effect with gender and duration. When examining profile similarity, results from the average discrepancy and ICC confirmed that couples with greater differences report better relationship quality.

Couples with greater discrepancy of conscientiousness perceive better relationship quality in the long term. The reason for this may be the social role of segmentation, which highlights the necessity of complementation in a smoothly functioning relationship (Eagly et al., 2000). This phenomenon is especially observed in organizations in which conscientious complementation enhances interaction quality (Chuang and Hsu, 2013). In addition, couple's relationship quality increases with an increase in openness discrepancy. Research has indicated that closed individuals are more inclined to seek open partners to balance themselves, enhance the stability of the relationship, and obtain comfort (Gurtman, 1995). Thus, complementary openness could help enhance couples' satisfaction levels. Zhou et al. (2017) found that, for Chinese dating young adults, openness in complementary matching dyads corresponds to better relationship quality than openness in moderate matching dyads.

When focusing on profile similarities, significant determinants can be observed in average discrepancy and ICC indices. In contrast to major empirical results showing that similarity positively predicted relationship quality, this study supported the complementary hypothesis (Moen et al., 2001; Shiota and Levenson, 2007).

We should consider differences between Chinese and Western cultures. Wu (2010) proposed that, in a collectivistic society, people attach less importance to personality similarity because personality is regarded as changeable and people are driven to adjust to the needs of others. In Wu, similarities in Big Five personality traits enhanced the relationship quality of American participants, but this did not apply to Chinese couples. This partly explained why complementarity is praised in Chinese. What is more, dating and married couples vary in their perceptions of partner selection, conflict resolution, and mutual communication. For purposes of companionship and self-growth, dating individuals are attracted to people with with characteristics different from their own (Blackwell and Lichter, 2004).

Another important finding was the gradual rise of the discrepancy effect corresponding to the length of the relationship. During the initial relationship stage, couples' main task is to establish trust and construct a stable satisfying relationship structure. To accelerate the pace and lay a solid foundation, discrepancy is less appreciated. Furthermore, individuals are more likely to be deceived by beliefs regarding an ideal mate and their partners' attractiveness, leading to less importance on actual discrepancy. With a generally well-established relationship, the next task is stability. Couples need to divide responsibilities and roles in specific domains, which may help them boost efficiency and avoid conflict. As a result, discrepancy is more appreciated in the long term (Shiota and Levenson, 2007; Simpson et al., 2015).

Profile similarity ICC also showed an asymmetry influence for males and females: Males enjoy better relationship quality when their partner is more similar in terms of personality. This result suggests the importance of viewing the meaning of profile similarity from both parties' perspectives, which encouraged us to distinguish the effect of similarity and complementarity in predicting different genders' perceived relationship quality in future studies.

## Implications

The results highlighted the importance of discriminating the effects of gender, relationship duration, and personality traits with respect to early-stage romantic relationships. Individual personality affects not only individuals' perceived relationship quality but also partners' satisfactory intimacy. Additionally, the study provides empirical support for the complementary hypothesis.

Our results highlight the joint effect of gender, relationship duration, and personality (including actor effect, partner effect, and similarity effect) and give us new insights into the personality effect's impact on dating relationship quality, which varied across gender and relationship duration. Thus, our results also have some practical implications insofar as they offer a new framework for understanding poor relationship quality by locating the problem from the perspective of the interaction of relationship duration and personality similarity.

## Limitations and further research

This study took young Chinese dating couples as research targets and examined the impact of personality and similarity on relationship satisfaction. There are some limitations that follow-up studies should improve upon.

First, although the results showed that there are complementary relationships in couple's personality, the unique trait combinations within couples are still unknown. As a next step, we would look into the dyad matching patterns rather than the similarity tendency of the couple (e.g., interactions between actor and partner's personality traits).

Furthermore, we treated relationship quality as a global unit, ignoring its components. Facing the distinct interpretations of romantic relationships in Chinese culture, future studies should deeply explore relationships' components, such as commitment, trust, and passion, to fully identify the mechanism of relationship quality.

In addition, the reliability of the Mini-IPIP used for personality measurement in this study was lower compared to that achieved in English samples (Donnellan et al., 2006). Meanwhile, the results might be less compelling than advertised due to a lack of correction for multiple testing correction (Cramer et al., 2016). We should be cautious in interpreting the results. Therefore, more studies should be done to confirm the conclusion.

## Ethics statement

This study was carried out in accordance with the recommendations of Ethics Committee of Institute of Psychology, Chinese Academy of Sciences with written informed consent from all subjects. All subjects gave written informed consent in accordance with the Declaration of Helsinki. The protocol was approved by the Ethics Committee of Institute of Psychology, Chinese Academy of Sciences.

## Author contributions

YZ conducted the analyses, interpreted the data, and drafted the manuscript; KW participated in the study's conception, design, and coordination and performed the measurement; SC participated in the study's coordination and performed the measurement; JZ helped in the design of the study and helped to draft the manuscript. MZ conceived of the study, and participated in its design and coordination and helped to draft the manuscript. All authors read and approved the final manuscript.

### Conflict of interest statement

The authors declare that the research was conducted in the absence of any commercial or financial relationships that could be construed as a potential conflict of interest.
